# CDC42-interacting protein 4 promotes metastasis of nasopharyngeal carcinoma by mediating invadopodia formation and activating EGFR signaling

**DOI:** 10.1186/s13046-016-0483-z

**Published:** 2017-01-28

**Authors:** Dong-Fang Meng, Ping Xie, Li-Xia Peng, Rui Sun, Dong-Hua Luo, Qiu-Yan Chen, Xing Lv, Lin Wang, Ming-Yuan Chen, Hai-Qiang Mai, Ling Guo, Xiang Guo, Li-Sheng Zheng, Li Cao, Jun-Ping Yang, Meng-Yao Wang, Yan Mei, Yuan-Yuan Qiang, Zi-Meng Zhang, Jing-Ping Yun, Bi-Jun Huang, Chao-Nan Qian

**Affiliations:** 1 State Key Laboratory of Oncology in South China; Collaborative Innovation Center for Cancer Medicine, Sun Yat-Sen University Cancer Center, Guangzhou, 510060 China; 2 Department of Pathology, Sun Yat-Sen University Cancer Center, Guangzhou, 510060 China; 3 Department of Nasopharyngeal Carcinoma, Sun Yat-Sen University Cancer Center, Guangzhou, 510060 China; 4 Radiotherapy Department, Affiliated Cancer Hospital of Guangzhou Medical University, Guangzhou, 510095 China

**Keywords:** NPC, CIP4, N-WASP, Invadopodia formation, EGFR/ERK/MMP-2 axis, Extracellular matrix degradation

## Abstract

**Background:**

Nasopharyngeal carcinoma (NPC) is a common malignancy in Southern China and Southeast Asia. In this study, we investigated the functional and molecular mechanisms by which CDC42-interacting protein 4 (CIP4) influences NPC.

**Methods:**

The expression levels of CIP4 were examined by Western blot, qRT-PCR or IHC. MTT assay was used to detect the proliferative rate of NPC cells. The invasive abilities were examined by matrigel and transwell assay. The metastatic abilities of NPC cells were revealed in BALB/c nude mice.

**Results:**

We report that CIP4 is required for NPC cell motility and invasion. CIP4 promotes the activation of N-WASP that controls invadopodia formation and activates EGFR signaling, which induces downstream MMP2 (matrix metalloproteinase 2) upregulation. In addition, CIP4 could promote NPC metastasis by activating the EGFR pathway. In nude mouse models, distant metastasis was significantly inhibited in CIP4-silenced groups. High CIP4 expression is an independent adverse prognostic factor of overall survival (OS) and distant metastasis-free survival (DMFS).

**Conclusion:**

We identify the critical role of CIP4 in metastasis of NPC which suggest that CIP4 may be a potential therapeutic target of NPC patients.

## Background

Nasopharyngeal carcinoma (NPC) is one of the most common malignancies in southern China and Southeast Asia [1, 2]. The standard treatment modality for NPC is radiotherapy and platinum-based chemotherapy [3–5]. Significant improvements in therapeutic efficacy have been achieved with the extensive application of intensity-modulated radiotherapy (IMRT) together with concurrent chemotherapy [6, 7]. Distant metastasis is the main reason of treatment failure [8]. However, the molecular mechanisms underlying NPC metastasis remain poorly understood.

Metastasis is a complex series of steps in which cancer cells leave the original tumor and spread to other organs via the bloodstream, lymphatic system, or body cavities [9]. To move toward other organs, cancer cells must extend their plasma membrane forward at the front, forming the leading edge of the cell. Cells extend four different plasma membrane protrusions at the leading edge: lamellipodia, filopodia, podosomes and invadopodia [10–12]. These structures uniquely contribute to cellular motility depending on specific circumstances [12]. Invadopodia are protrusions that allow focal degradation of the extracellular matrix to facilitate invasion through the tissues. Invadopodium extension in three dimensions (3D) requires force driven by actin polymerization. Demonstration of invadopodia is typically performed on two-dimensional (2D) surfaces coated with extracellular matrix proteins, where the invadopodia are present on the ventral surface [13–15]. Invadopodia degrade the extracellular matrix and require the delivery of vesicles containing matrix-degrading proteases, particularly membrane type 1 metalloprotease (MT1-MMP) from the cellular plasma to invadopodial tip. These vesicles are targeted to invadopodia by the vesicle-tethering exocyst complex [16].

In mammals, the TOCA family (also named F-BAR proteins) includes three members: TOCA-1 (Transducer of CDC42-dependent actin assembly), CIP4 (CDC42-interacting protein 4), and FBP17 (formin-binding protein 17). CIP4 is implicated in clathrin-mediated endocytosis (CME), during which it senses and promotes membrane curvature through its F-BAR domain and binds to key regulators of actin dynamics (e.g., the nucleation promoting factor N-WASP) and endocytosis (e.g., dynamin) through their SH3 domain [17, 18]. Furthermore, CIP4 acts as an effector of the small GTPase CDC42 that promotes cell migration in breast cancer [19, 20].

Here, we demonstrate that by regulating invadopodia formation, assembly and extracellular matrix (ECM) degradation, CIP4 controls cell migration and invasion in response to EGFR signaling. We further demonstrate that CIP4 knock-down (KD) had no overt effect on tumor growth, but impaired the ability of distant metastasis in mouse xenograft models. Consistently, CIP4 expression is increased in NPC compared with nasopharyngeal mucosa. Evaluating the expression of CIP4 in primary tumors from 169 NPCs also revealed that high CIP4 protein levels correlate with worse overall survival (OS) and distant metastasis-free survival (DMFS) in NPC patients.

## Methods

### Cell culture, cellular growth curve, and colony-formation assays

The human nasopharyngeal carcinoma cell lines 5-8F and S18 were maintained in Dulbecco’s modified Eagle’s medium supplemented with 10% FBS at 37 °C and 5% CO_2_.

Cellular growth curves were plotted by using the cellular viability values assessed by the MTT method (Cell Titer 96 Aqueous One Solution Cell Proliferation Assay solution; Sigma). Briefly, 1000 cells/200 μl of medium were seeded into a 96-well plate (Corning) and cultured under normal conditions. At various time points after seeding, the cells in each well were stained with MTT (Sigma, M2128) for 3 h. Then, medium was discarded, and 200 μl of DMSO was added to each well and incubated for 10 min, and the OD490 was determined with a microplate reader.

For the colony-formation assays, 500 cells/2 ml were seeded into a 6-well plate (Corning). After 10 days, the cells were washed with phosphate-buffered saline (PBS), fixed with methanol for 15 min at room temperature, and stained with 1% crystal violet for 20 min. The colonies were counted. All experiments were independently repeated at least three times.

### RNA isolation and real-time quantitative reverse-transcription PCR (qPCR)

Total RNA was extracted from cultured cell lines using TRIzol reagent (Invitrogen) and subjected to reverse transcription using a cDNA Synthesis Kit (Thermo, K1622). Real-time qPCR was performed using a SYBR FAST Universal qPCR Kit (KAPA, KK4602). The relative expression levels of the target genes were calculated as two power values of ΔCt (the Ct of GAPDH or CIP4 minus the Ct of the target gene). The sequences of the PCR primers used for amplification were as follows:

- GAPDH forward, 5′- GTCTCCTCTGACTTCAACAGCG -3′;
- GAPDH reverse, 5′- ACCACCCTGTTGCTGTAGCCAA -3′;
- CIP4 forward, 5′- CGAATATGCGGCTCAACTGCAG -3′;
- CIP4 reverse, 5′- CCTGCGTTCATCCATGTCTTGG -3′.

### Small interfering RNA transfection

The negative control small interfering RNA (NC) was purchased from RIBOBIO, and siRNA sequences targeting human CIP4 are 5′- GCATGAAGGTGGCTGCAAA-3′(si#1) and 5′- CCGAAGTGGAACAGGCTTA -3′(si#2). Transient transfections of NPC cells were performed as described previously using the Lipofectamine RNAiMAX Reagent (Invitrogen) protocol. Briefly, 60 pmol siRNA was mixed with Opti-MEM Medium (Invitrogen) and incubated at room temperature for 15 min. Then, the mixture was added to the cells.

### Lentiviral transduction studies

Cell lines stably expressing CIP4 short hairpin RNA (shRNA) or a negative control shRNA were purchased from FulenGen Co. Ltd. (Guangzhou, China). Lentiviruses were produced by 293T cells with one of the shRNA using X-tremeGENE DNA transfection reagents (Roche). Infectious lentiviruses were harvested 48 h after transfection and filtered through 0.45 mm filter (Millipore, Bedford, MA). Cells were transduced with lentiviruses CIP4 shRNA or negative control shRNA and then cultured in medium containing 2 mg/ml puromycin (Sigma) for 3 days for selection. CIP4 knockdown efficiency was determined by immunoblotting.

### Immunoblotting

Immunoblotting was performed using the standard protocol. The primary antibodies, including rabbit anti-human CIP4 polyclonal antibody (Proteintech), rabbit anti-human N-WASP polyclonal antibody (Proteintech), rabbit anti-human phospho-N-WASP polyclonal antibody (Abcam), rabbit anti-human MMP2 polyclonal antibody (Cell Signaling Technology), rabbit anti-human MMP9 polyclonal antibody (Cell Signaling Technology), rabbit anti-human ERK1/2 polyclonal antibody (Cell Signaling Technology), rabbit anti-human phospho-ERK polyclonal antibody (Cell Signaling Technology), rabbit anti-human EGFR polyclonal antibody (Cell Signaling Technology), rabbit anti-human phosphor-EGFR polyclonal antibody (Cell Signaling Technology), rabbit anti-human AKT1 polyclonal antibody (Cell Signaling Technology), rabbit anti-human phospho-AKT polyclonal antibody (Cell Signaling Technology) and β-actin polyclonal antibody (Cell Signaling Technology) were used at a dilution of 1:1000.

### ECM degradation assay

For ECM degradation assay, glass-bottom dishes were coated with Gelatin From Pig Skin, Oregon Green® 488 Conjugate (Invitrogen) and then treated with 0.5% glutaraldehyde as described earlier [21–23]. Cells were cultured on these glass-bottom dishes in DMEM, fixed and stained with anti-cortactin antibody or Rhodamine Phalloidin (Cytoskeleton). Fluorescent images were obtained using a laser scanning confocal imaging system (OLYMPUS FV1000). Cells in which dot-like degradation of Alexa-gelatin was observed were judged as positive for invadopodia.

### Migration and invasion assays

Migration assays were conducted with Biocoat without Matrigel (Corning. Life sciences), and invasion assays were performed with Biocoat with Matrigel (Corning. Life sciences) following the manufacturer’s instructions. The harvested Biocoats were then stained with crystal violet, and invaded cells were counted under a microscope. Both experiments were repeated independently three times.

### Animal experiments

Female athymic mice (Beijing Charles River Laboratory Animal Center) were purchased at 4–5-weeks-of-age and maintained under a specific pathogen-free environment. All animal experiments were approved by the Institutional Animal Care and Use Committee of the Sun Yat-Sen University Cancer Center.

For the tumor xenograft experiments, the tumor cells (1 × 10^6^ cells/tumor in 100 μl DMEM) were intravenously injected through the tail vein of mice. Distant metastases in lungs were assessed and counted after 5 weeks when mice were sacrificed. Lungs and livers were excised and embedded in paraffin for further study.

The spontaneous lymph node (LN) metastasis experiments were conducted as previously reported [24–26]. Briefly, 2 × 10^5^ cells in 20 μl DMEM were subcutaneously injected into the footpad of the left hind limb of each mouse to generate a primary tumor. After 4 weeks, the experiments were terminated, and the popliteal LNs of the left hind feet were isolated and preserved in RNAlater solution (Invitrogen). The primary tumor weight was measured and calculated by subtracting the weight of the contralateral foot without the tumor from the weight of the foot carrying the tumor. LNs were homogenized in TRIzol for total RNA extraction using the Bullet Blender (Next Advance). Reverse transcription and real-time PCR were performed to assess metastasis using specific primers for human HPRT, which do not cross-react with the corresponding mouse gene [27]. The following human and mouse primers were used:

- HPRT forward: 5′-TTCCTTGGTCAGGCAGTATAATCC-3′;
- HPRT reverse: 5′-AGTCTGGCTTATATCCAACACTTCG-3′;
- ACTB (universal for human and mouse) forward:
- 5′-CAATGAGCTG CGTGTGGC-3′;
- ACTB (universal for human and mouse) reverse:
- 5′-CGTACATGGC TGGGGTGTT-3′.

### Human tissue samples

To compare the mRNA expression levels of CIP4 among different stages of NPC development, 19 non-cancerous nasopharyngeal mucosa and 15 primary NPCs were obtained at the Department of Nasopharyngeal Carcinoma, Sun Yat-sen University Cancer Center (SYSUCC). In total, 169 formalin-fixed and paraffin-embedded NPC specimens were obtained from patients at SYSUCC pathologically diagnosed between February 2006 and December 2009. The 169 cases of NPC with sufficient follow-up data qualified for analyses after immuno-histochemical (IHC) staining for CIP4. All human tissue samples were obtained with patient consent and the approval of the Institutional Clinical Ethics Review Board at SYSUCC.

In IHC analysis of CIP4, the paraffin-embedded slices were deparaffinized, rehydrated, and blocked in 5% bovine serum albumin (BSA) at room temperature for 20 min. The samples were incubated with rabbit polyclonal antibody against CIP4 (ab108313, Abcam) at a dilution of 1:100 at 4 °C overnight followed by horseradish peroxidase (HRP) anti-rabbit immunoglobulin at a concentration of 1:100 for 30 min at 37 °C. The primary antibodies were detected with 3, 3-diaminobenzidine substrate visualization and counterstaining with hematoxylin (GTVision III Detection System/Mo & Rb). For each tumor, we determined a proportion score and an intensity score. Cytoplasmic and membranous staining intensity were categorized as follows: absent staining as 0, weak as 1, moderate as 2, and strong as 3. The percentage of stained cells was categorized as no staining = 0, 1–10% of stained cells = 1, 11–50% = 2, 51–80% = 3, and 81–100% = 4. The proportion and intensity were then multiplied to produce a total score ranging from 0 to 12. The median score of CIP4 (score = 4) was used as the cutoff value to divide the patients into the high (> median) and low (≤ median) CIP4 expression groups.

### Statistical analysis

Student’s *t*-test was used to compare two independent groups of data. The median IHC staining score was used as a cut-off value to divide the patients into low and high CIP4 expression groups. Chi-squared tests were applied to analyze the relationship between CIP4 expression and clinicopathological status. The significance of several variables for survival was analyzed using the Cox regression model in a multivariate analysis. *P*-value < 0.05 was considered statistically significant in all cases.

## Results

### CIP4 is highly expressed in NPC tissues and is associated with poor prognosis

**Fig. 1:**
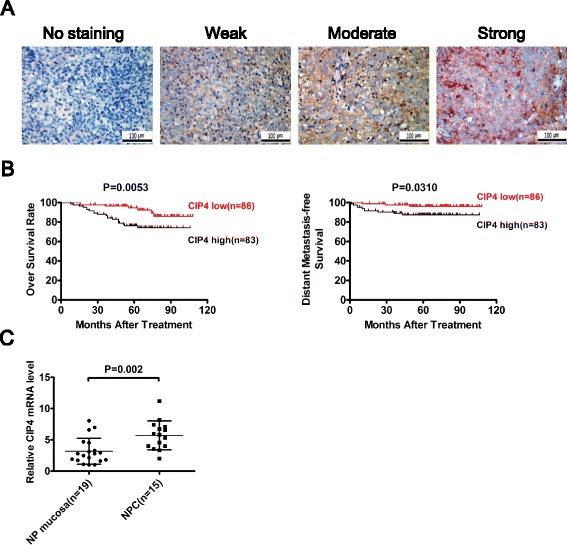
High CIP4 expression correlates with shorter overall survival and distant-metastasis-free survival in NPC patients. **a** Levels of CIP4 protein expression in NPC tissues are shown under high magnifications microscopy. **b** Kaplan-Meier analysis indicates upregulation of CIP4 was significantly associated with poorer overall survival and distant metastasis-free survival of NPC patients (*p* = 0.0053, *p* = 0.0310, respectively). **c** CIP4 mRNA expression in the NPC tissues and non-cancerous nasopharyngeal (NP) mucosa tissues detected by qPCR

To investigate the underlying clinical significance of CIP4, the CIP4 expression level with clinicopathological features in 169 NPCs (informative IHC cases) was analyzed (Fig. 1a). High CIP4 expression was significantly associated with M stage and prognosis (Table 1). Multivariate analyses of different prognostic parameters revealed that high CIP4 expression was an independent, unfavorable prognostic indicator for OS and DMFS (Table 2). In the Kaplan-Meier analysis, OS and DMFS were increased for patients with low CIP4 expression compared with those with high CIP4 expression (Fig. 1b). CIP4 mRNA levels were also increased in NPC tissues compared with nasopharyngeal mucosa (Fig. 1c). These data collectively demonstrate a close correlation between CIP4 expression level and poor patient outcomes, implying an important role for CIP4 in NPC progression.

**Table 1:** Association between expression of CIP4 and clinicopathological characteristics in 169 NPC patients

Clinical factor	Cases (*n* = 169)	CIP4 expression		*P* value
		High	Low	
		(*n* = 83)	(*n* = 86)	
Gender				
Male	128	63	65	0.961
Female	41	20	21	
Ages (years)				
< 45	79	39	40	0.951
≥ 45	90	44	46	
T stage				
T1-2	49	22	27	0.484
T3-4	120	61	59	
N stage				
N0-1	89	41	48	0.404
N2-3	80	42	38	
M stage				
M0	160	75	85	**0.035**
M1	9	8	1	
Clinical stage				
I-II	17	6	11	0.229
III-IV	152	77	75	
WHO histological classification Type 2				
Differentiated	8	2	6	0.300
Undifferentiated	161	81	80	
Local-regional relapse				
No	166	81	85	0.975
Yes	3	2	1	
Distant metastasis				
No	156	73	83	0.072
Yes	13	10	3	
Progression				
No	147	66	81	**0.005**
Yes	22	17	5	
Death				
No	142	63	79	**0.005**
Yes	27	20	7	

Statistical significance (*p* < 0.05) is shown in bold and italic

**Table 2:** Univariate and multivariate analyses of different prognostic parameters in NPC patients

Variables	Univariate analysis			Multivariate analysis		
	HR	Cl	*P*	HR	Cl	*P*
OS						
Gender	2.763	0.832–9.181	0.097	…	…	…
Age	2.841	1.198–6.734	**0.018**	5.148	1.933–13.707	**0.001**
T stage	1.317	0.554–3.129	0.533	…	…	…
N stage	1.745	0.810–3.761	0.155	…	…	…
M stage	9.441	3.769–23.647	**<0.001**	14.195	4.642–43.407	**<0.001**
Clinical stage	24.271	0.134–4382.908	0.229	…	…	…
CIP4	1.137	1.043–1.240	**0.004**	1.111	1.015–1.216	**0.022**
DMFS						
Gender	4.019	0.523–30.911	0.181	…	…	…
Age	1.451	0.475–4.436	0.514	…	…	…
T stage	0.666	0.218–2.037	0.476	…	…	…
N stage	1.908	0.624–5.832	0.257	…	…	…
M stage	13.355	4.058–43.955	**<0.001**	8.565	2.495–29.401	**0.001**
Clinical stage	23.874	0.011–53961.801	0.421	…	…	…
CIP4	1.199	1.046–1.374	**0.009**	1.159	1.006–1.336	**0.041**

Abbreviations: *OS* overall survival, *DMFS* distant metastasis-free survival, *CI* confidence interval, *HR* hazard ratio. Statistical significance (*p* < 0.05) is shown in bold and italic

### Knocking-down CIP4 inhibits the migration and invasion of highly metastatic NPC cells without influencing general cell growth or contact-independent cell growth

**Fig. 2:**
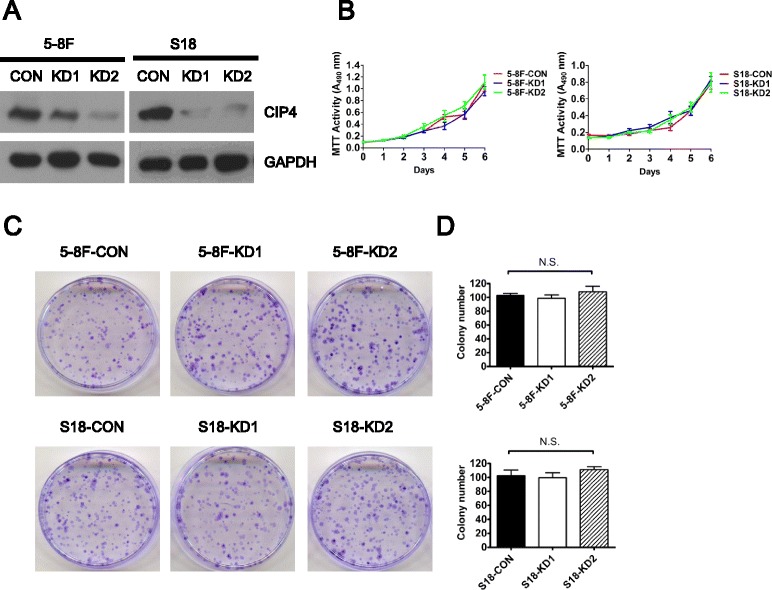
Suppression of CIP4 expression has no effect on growth in NPC cells in vitro. **a** Decreased expressions of CIP4 were respectively confirmed by Western blotting in CIP4-silenced 5-8F and S18 cells compared with scrambled shRNA control cells. **b** Cell growth rates between CIP4-silenced and scrambled shRNA control cells were compared by MTT assay. **c** and **d** The colony formation assays were performed to determine the effect of growth in CIP4 knockdown and control cells. N.S. means no significance. The data are presented as the mean ± S.D. (from triplicates)

To further confirm whether CIP4 influences cell mobility in migration and invasion without affecting tumor formation, CIP4 was knocked down in two highly metastatic cell lines (5-8F and S18) via RNA interference (RNAi) with two shRNAs targeting CIP4 (CIP4-KD1 and CIP4- KD2). A scramble shRNA was used as a control (CIP4-CON). Western blotting revealed a significant reduction in CIP4 protein levels (Fig. 2a).

Functional assays of cell growth curves and colony formation revealed that the NPC cell growth rate and contact-independent cell growth were not significantly altered in CIP4 KD cells compared with control cells (Fig. 2b, c and d).

### CIP4 promotes NPC cell migration and invasion in vitro

**Fig. 3:**
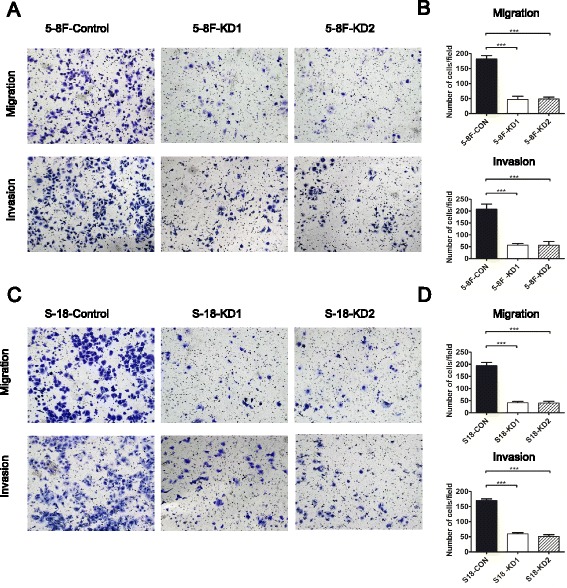
Suppression of CIP4 expression inhibits the migration and invasion of highly metastatic NPC cells in vitro. **a** and **c** Silencing CIP4 could inhibit cell migration and cell invasion in 5-8F and S18 cells compared with scrambled shRNA control cells. **b** and **d** Columns, average of three independent experiments; *Bars*, S.D. ***P < 0.001

To examine the causal role of CIP4 in NPC cell motility, we used migration assays to evaluate cell migration in CIP4 control and KD cells. CIP4-KD cells exhibited reduced migration compared with controls (Fig. 3a and c). Next, we compared the effects of CIP4-KD on the invasive potential of 5-8F and S18 cells using Matrigel-coated transwell chambers. Interestingly, both 5-8F and S18 KD cells exhibited severe defects in cell invasion compared with their respective controls (Fig. 3b and d). Together, these results suggest that CIP4 is a positive regulator of NPC cell migration and invasion through the ECM.

### CIP4 regulates invadopodia assembly through activation of N-WASP

**Fig. 4:**
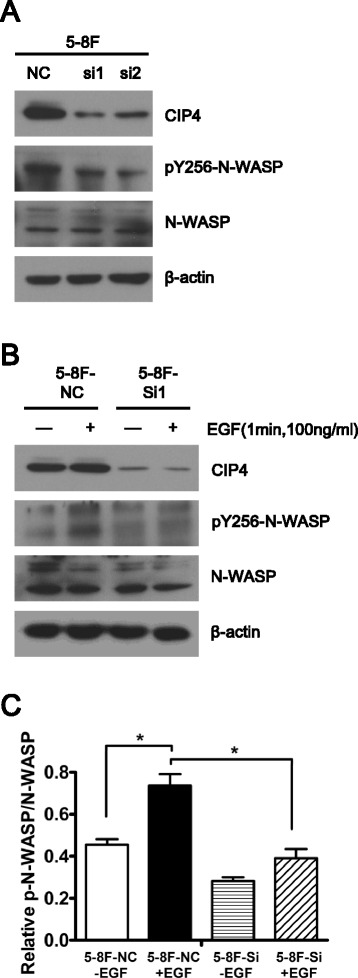
CIP4 promotes the EGF-dependent activation of N-WASP in NPC cells. **a** 5-8F cells were transfected with control or CIP4-siRNA and whole-cell lysates were immunoblotted for phospho-N-WASP (Y256), total N-WASP and CIP4 at 72 h after transfection. Blots were reprobed for β-actin. **b** NPC cells transfected with control or CIP4-siRNA were treated with EGF for 1 min, lysed and probed for phospho-N-WASp (Y256) and then reprobed for total N-WASp and CIP4. Blot is representative of three independent experiments. **c** densitometric quantification of immunoblot in b

The Arp2/3 complex and neural Wiskott–Aldrich syndrome protein (N-WASP; encoded by WASL) are essential components of invadopodia, which regulate actin polymerization in the early phase of invadopodium assembly [28]. Lorenz and colleagues previously measured and directly imaged N-WASP activity in vivo by using FRET microscopy and observed that N-WASP was involved in the cytoskeleton reorganization of invadopodia of migrating carcinoma cells. N-WASP can be activated by many upstream factors, including Cdc42, PIP2, or phosphorylation, and it is likely that different cell responses are regulated by different upstream activators [29]. Because CDC42 interacts with N-WASP and facilitates the nuclear translocation of EGFR [30], we investigated the role of CIP4 EGF-induced N-WASP activation. N-WASP phosphorylation was decreased in CIP4-silenced cells compared with control cells (Fig. 4a). Previous research showed a rapid, transient increase in acceptor photobleaching fluorescence resonance energy transfer (apFRET) efficiency between CIP4 and N-WASP after EGF stimulation in the fluorescence resonance energy transfer assay [20]. In the presence of EGF, control 5-8F cells increased the phosphorylation of N-WASP at 1 min (Fig. 4b). Knockdown of CIP4 decreased the basal level of N-WASP phosphorylation in 5-8F cells (Fig. 4c). These results suggest that CIP4 significantly altered invadopodia assembly by affecting the level of activated N-WASP.

### CIP4 has important functions in invadopodia formation and ECM degradation

**Fig. 5:**
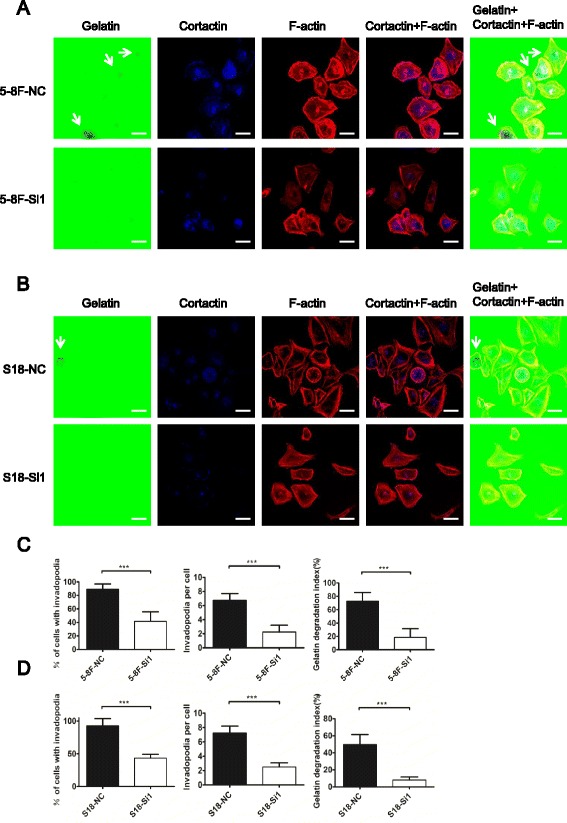
CIP4 plays an important role in invadopodia formation in NPC cells. **a** and **b** 5-8F cells and S18 cells transfected with control or CIP4 siRNA were plated on glass-bottom dishes coated with Gelatin From Pig Skin, Oregon Green® 488 Conjugate and cultured for 20 h before being stained for endogenous CIP4 and F-actin. The cells were fixed and stained with anti-cortactin antibody (*blue*) and F-actin (*red*). The *arrowheads* indicate the position of extracellular matrix (ECM)-degrading invadopodia. *Scale bar*, 30 μm. **c** and **d** Gelatin degradation areas were counted and measured, normalized for cell number and averaged over replicates from three independent experiments. The data are expressed as the mean ± S.D. of three independent experiments. ***, *P* < 0.001

N-WASP is an actin-regulatory protein associated with invadopodium markers, including ARP2/3 and cortactin. These proteins then form a complex with the small GTPase CDC42 [31]. To determine whether CIP4 played an important role in invadopodia formation, NPC cells were plated on glass-bottom dishes of Oregon Green® 488 Conjugate-labeled gelatin and incubated overnight to allow invadopodia formation. As shown in Fig. 5a and b, the formation of invadopodia was visualized by co-immunostaining cells for filamentous actin (F-actin) using fluorescently conjugated phalloidin (red) and invadopodium-associated protein cortactin (blue). Dot-like ECM degradation (loss of green color) under the cell was also observed (see arrowhead).

To examine the role of CIP4 in invadopodia formation by NPC cells, we quantified the percentage of cells with invadopodia and the distribution of the number of invadopodia per cell after treatment knockdown with control or CIP4 siRNA. We observed a significantly reduced percentage of cells with invadopodia and fewer invadopodia per cell in CIP4-siRNA-treated cells. To evaluate the size and function of invadopodia, we quantified the area of gelatin degradation per cell and found significantly less gelatin degradation after CIP4 silencing (Fig. 5c and d).

### CIP4 regulates EGFR signaling and promotes MMP-2 expression in NPC cells

**Fig. 6:**
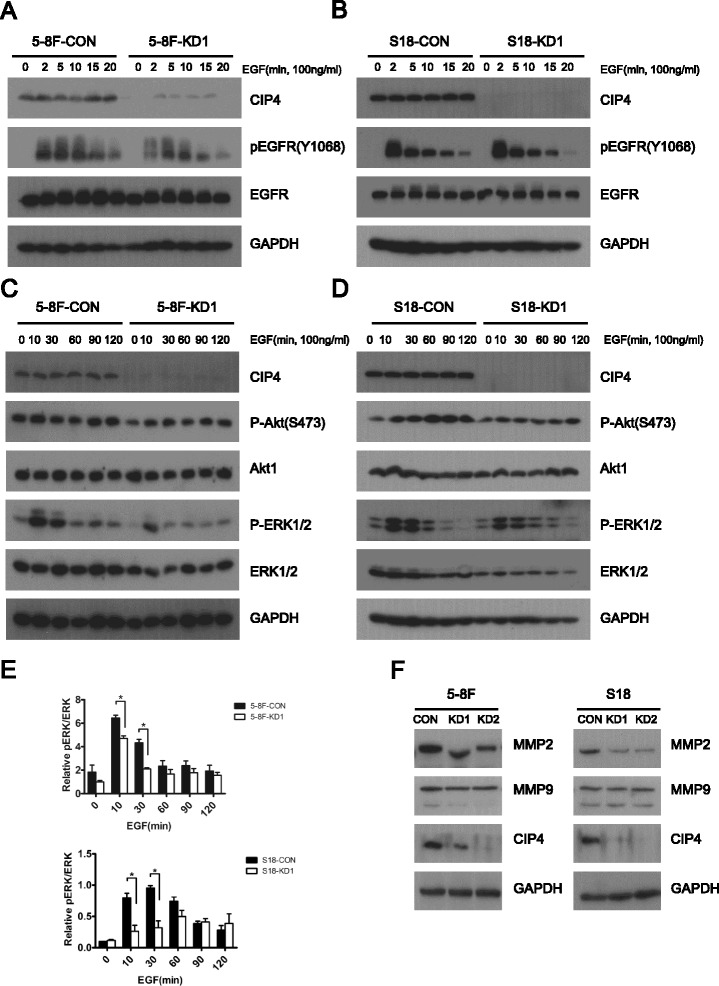
CIP4 modulates EGFR signaling and promotes MMP-2 expression in NPC cells. **a** and **b** Serum-starved 5-8F and S18 Control and KD1 cells were treated with EGF (100 ng/ml) for 0–20 min. Lysates were subjected to IB with the antibodies indicated on the *right*. **c** and **d** Serum-starved 5-8F and S18 Control and KD1 cells were treated with EGF (100 ng/ml) for 0–120 min. Lysates were subjected to IB with the indicated antibodies. **e** Densitometry was performed, and relative phospho-Erk levels are shown in the graph below (mean ± S.D.). *, *P* < 0.05. **f** IB analysis of MMP-2 and MMP-9 in 5-8F and S18 Control and KD1 cell lysates. GAPDH was used as a loading control

TOCA family members control early events of epidermal growth factor receptor (EGFR) clathrin-mediated endocytosis (CME) and trafficking [17, 32], and EGFR is widely expressed in NPC [33]. To assess the effects of CIP4 on EGFR signaling in NPC cells, we performed a 20-min time course of EGF treatment. EGF treatment of CIP4 CON and KD cells led to rapid phosphorylation of EGFR (pEGFR; Y1068) that was sustained throughout the time course. CIP4 KD cells exhibited no overt defects in EGF-induced pEGFR levels compared with control cells (Fig. 6a and b).

To address whether CIP4 regulates EGFR signaling to downstream pathways, we profiled EGF-induced phosphorylation of the activation loop sites (S473) in Akt (pAkt) and Erk kinases (pErk) in CIP4 CON and KD cells. EGF-induced phosphorylation of Akt (S473) did not differ in CIP4 CON and KD cells (Fig. 6c and d). In contrast, CIP4 KD resulted in a less sustained phosphorylation of Erk with EGF treatment (Fig. 6e).

The maturation process for invadopodia involves the recruitment and activation of multiple pericellular proteases that facilitate ECM degradation, such as zinc-regulated metalloproteases (matrix metalloprotease 2 (MMP2), MMP9, MT1-MMP) [34, 35]. Therefore, we investigated whether CIP4 KD effects the expression of MMPs. Immunoblotting revealed a significant reduction in MMP-2 but not MMP-9 in CIP4 KD cells (Fig. 6f). Taken together, these results suggest that CIP4 modulates the kinetics of EGFR signaling and promotes MMP-2 expression in NPC cells.

### CIP4 silencing impairs NPC metastasis in vivo

**Fig. 7:**
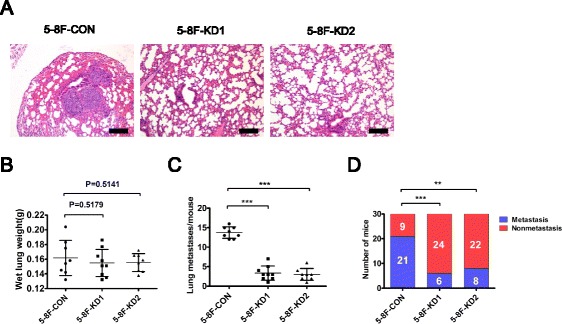
CIP4 promotes distant metastasis in vivo. **a** Histological image of lung metastasis in nude mice after tail vein injection of 5-8F cells. *Scale bar*, 500 μm. **b** The average wet lung weights of the mice were not difference between CIP4 KD and controls. **c** The numbers of lung metastases (mean ± S.D.) were counted and summarized. ***P < 0.001. **d** The metastasis rates of CIP4 knockdown 5-8F cells in vivo. The proportion of popliteal lymph node metastases was significantly reduced upon CIP4 silencing in 5-8F cells (*n* = 30 per group). *P* values were calculated using a chi-square test

To evaluate the effects of CIP4 on tumor metastasis in vivo, the same amount of shRNA-transfected cells (5-8F-shRNA-CIP4-1, 5-8F-shRNA-CIP4-2) and their control were injected into nude mice intravenously through the tail vein. After 6 weeks, the mice were euthanized, and metastatic lung nodules were counted (Fig. 7a). Although there was no difference in average tumor mass between CIP4 KD and controls (Fig. 7b), scoring of the numbers of metastases from hematoxylin and eosin (H&E)-stained lung tissue sections revealed a significant reduction in incidence compared with control mice (Fig. 7c). To extend these findings, we utilized a popliteal lymph node (LN) metastasis model. The spontaneous metastasis experiments indicate that the popliteal LN metastasis rate was significantly reduced from 70% (21/30) to 20% (6/30) or 26.7% (8/30) via suppression of CIP4 expression in NPC cells (Fig. 7d). Together these results corroborate the importance of CIP4 in the regulation of NPC tumor metastasis in vivo.

## Discussion

The main obstacle in the current clinical management of NPC is metastasis [36]. Given its high metastasis rate, NPC cell motility has been linked to the formation of different types of cellular membrane protrusions. Lamellipodia extend long distances through the extracellular matrix and pull cell through the tissues. In filopodia, actin polymerization directly pushes the cytomembrane forward [37–39]. Invadopodia deliver matrix-degrading metalloproteases to clear a path for cells through the extracellular matrix [40]. Our functional studies confirmed that silencing CIP4, the regulator of invadopodia, impaired MMPs-mediated degradation of collagen to generate the ECM tracks.

CIP4 is an F-BAR protein that regulates actin-based cell motility [30]. Roles of CIP4 in cell migration have been described in neuronal, B lymphoma cells and breast cancer [20, 41, 42]. However, we demonstrate the role of CIP4 in the regulation of invadopodia formation, cell-migration and cell-ECM degradation during NPC metastatic events in the present study. We show that inducible silencing of CIP4 results in defects in EGFR signaling and impaired motility and invasion of NPC cells. We also tested the effects of CIP4 silencing on NPC metastasis in tumor xenograft assays and observed a key role for CIP4 in NPC metastasis. Importantly, our study also profiled CIP4 expression in human nasopharyngeal carcinoma patients, which revealed links between high CIP4 levels and worse prognosis. Together with our findings in NPC models, these studies identify CIP4 as a key signaling hub in NPC metastasis.

N-WASP and Cdc42 are critical for the formation of invadopodia, which are specialized cytoskeletal structures that combine localized actin protrusion with matrix metalloproteinases (MMPs) secretion to degrade extracellular matrices and allow invasion [43, 44]. The formation of invadopodia requires the activation of the Cdc42–N-WASP pathway [45]. We found that CIP4 is fully indispensable in mediating the activation of a CDC42/N-WASP, a function likely fulfilled by the other members of the family. Instead, CIP4 is essential for the formation of invadopodia.

Since the majority of NPCs express high levels of EGFR, there has been considerable interest in testing EGFR signaling. Several previous studies have functionally linked CIP4 to EGFR trafficking and downstream signaling to pathways controlling cell motility and invasiveness [32, 46]. In the present study, we showed that silencing CIP4 in NPC cell lines resulted in impaired EGFR signaling to ERK, whereas high CIP4 expression promoted activation of the EGFR/ERK/MMP-2 axis in NPC cells. Others have also reported a role for CIP4 in promoting Src activation and Cadherin switching in mammary epithelial cells treated with EGF or TGFβ [32].

## Conclusions

In summary, CIP4 plays an important role in the promotion of NPC metastasis by mediating invadopodia formation and activating the EGFR pathway, which may lead to the identification of a new therapeutic target for distant metastasis of NPC.

## Abbreviations

apFRET: Acceptor photobleaching fluorescence resonance energy transfer
CIP4: CDC42-interacting protein 4
CIP4: CDC42-interacting protein 4
CME: Clathrin-mediated endocytosis
DMFS: Distant metastasis-free survival
ECM: extracellular matrix
FBP17: Formin-binding protein 17
H&E: Hematoxylin and eosin
IHC: Immuno-histochemical
IMRT: Intensity-modulated radiotherapy
MMP2: Matrix metalloproteinase 2
MT1-MMP: Membrane type 1 metalloprotease
NPC: Nasopharyngeal carcinoma
N-WASP: Wiskott–Aldrich syndrome protein
OS: Overall survival
RNAi: RNA interference
TOCA-1: Transducer of CDC42-dependent actin assembly
