# Erratum to: CDC42-interacting protein 4 promotes metastasis of nasopharyngeal carcinoma by mediating invadopodia formation and activating EGFR signaling

**DOI:** 10.1186/s13046-017-0503-7

**Published:** 2017-02-20

**Authors:** Dong-Fang Meng, Ping Xie, Li-Xia Peng, Rui Sun, Dong-Hua Luo, Qiu-Yan Chen, Xing Lv, Lin Wang, Ming-Yuan Chen, Hai-Qiang Mai, Ling Guo, Xiang Guo, Li-Sheng Zheng, Li Cao, Jun-Ping Yang, Meng-Yao Wang, Yan Mei, Yuan-Yuan Qiang, Zi-Meng Zhang, Jing-Ping Yun, Bi-Jun Huang, Chao-Nan Qian

**Affiliations:** 1 State Key Laboratory of Oncology in South China; Collaborative Innovation Center for Cancer Medicine, Sun Yat-Sen University Cancer Center, Guangzhou, 510060 China; 2 Department of Pathology, Sun Yat-Sen University Cancer Center, Guangzhou, 510060 China; 3 Department of Nasopharyngeal Carcinoma, Sun Yat-Sen University Cancer Center, Guangzhou, 510060 China; 4 Radiotherapy Department, Affiliated Cancer Hospital of Guangzhou Medical University, Guangzhou, 510095 China

## Erratum

In the original publication of this article [1], the corresponding author’s email address was listed incorrectly. The correct email address for the corresponding author is qianchn@sysucc.org.cn.
